# Life history and dynamics of a platypus (*Ornithorhynchus anatinus*) population: four decades of mark-recapture surveys

**DOI:** 10.1038/srep16073

**Published:** 2015-11-05

**Authors:** Gilad Bino, Tom R. Grant, Richard T. Kingsford

**Affiliations:** 1 Centre for Ecosystem Science, School of Biological, Earth & Environmental Sciences, University of New South Wales, Sydney 2052 NSW, Australia

## Abstract

Knowledge of the life-history and population dynamics of Australia’s iconic and evolutionarily distinct platypus (*Ornithorhynchus anatinus*) remains poor. We marked-recaptured 812 unique platypuses (total 1,622 captures), over four decades (1973–2014) in the Shoalhaven River, Australia. Strong sex-age differences were observed in life-history, including morphology and longevity. Apparent survival of adult females (Φ = 0.76) were higher than adult males (Φ = 0.57), as in juveniles: females Φ = 0.27, males Φ = 0.13. Females were highly likely to remain in the same pool (adult: *P* = 0.85, juvenile: *P* = 0.88), while residency rates were lower for males (adult: *P* = 0.74, juvenile: *P* = 0.46). We combined survival, movement and life-histories to develop population viability models and test the impact of a range of life-history parameters. While using estimated apparent survival produced unviable populations (mean population growth rate r = −0.23, extinction within 20 years), considering residency rates to adjust survival estimates, indicated more stable populations (r = 0.004, p = 0.04 of 100-year extinction). Further sensitivity analyses highlighted adult female survival and overall success of dispersal as most affecting viability. Findings provide robust life-history and viability estimates for a difficult study species. These could support developing large-scale population dynamics models required to underpin a much needed national risk assessment for the platypus, already declining in parts of its current distribution.

The platypus (*Ornithorhynchus anatinus*) is one of only five extant species of egg-laying mammals and the only species within the family *Ornithorhynchidae*^1^. It is one of the most evolutionarily distinct mammals on Earth, belonging to a subclass separated from all other living mammals^2^,^3^,^4^, making it of exceptional scientific value and an irreplaceable component of Australian and global biodiversity. Except for in the far north, this endemic species occurs along the margins of the eastern Australian mainland and in Tasmania and adjacent King Island, with a small introduced population on Kangaroo Island. Genetic analyses indicate three natural sub-populations: northern Queensland, Tasmania/King Island, and the rest of mainland Australia^5^,^6^,^7^. It lives and breeds mainly in permanent reaches of streams but also in some lakes and wetlands, from which juveniles disperse, following each breeding season.

Mounting evidence of recent local platypus population declines and extinctions highlight a species facing considerable risks^8^,^9^,^10^,^11^,^12^. Its range coincides with Australia’s most highly regulated and disrupted rivers^1^,^13^ and the species faces a range of threats resulting from human activities, including agriculture, forestry, mining, urbanisation and fragmentation by dams and other in-stream structures^14^,^15^,^16^; by-catch mortality in fishing gear^1^,^17^ and predation by foxes and feral dogs^1^,^18^,^19^. The platypus is “of least concern”, under current IUCN red listing^20^, but were identified recently as ‘near threatened’, given localised declines and extinctions in populations, particularly in the state of Victoria^21^. Despite increasing understanding, many aspects of the species’ biology, including its population dynamics remain relatively poor, reliant on few long-term studies investigating densities, reproduction, age structure and survival^22^,^23^,^24^. There are generally low recapture rates^22^,^25^, making reliable estimates of population sizes difficult. Lack of population estimates and trends have hindered assessment of threatening processes and assessment of the conservation status of the platypus. Given this, modelling of population dynamics of platypuses is rare with few robust estimates of survival and viability.

Globally, there is growing concern that extinction risk to common and widespread species is rapidly increasing, with little analyses or implementation of conservation assessment or actions. Instead, such assessments and actions are primarily focused on threatened species^26^. Collection of life history data, estimation of population viability and assessment of effects of threatening processes may be difficult for species like the platypus, despite their status as common or ubiquitous. Given increasing anthropogenic pressures on freshwater environments^27^,^28^, many more species, including those currently considered ‘common’, may be pushed beyond viable tipping points, accelerating decline and possibly resulting in extinction. The platypus is particularly at risk because of its relatively poor and diffuse information base, particularly on population dynamics^20^,^21^. Vital rates and population dynamics underpin risk assessments of species, providing evidence for effective conservation actions^29^. Effective conservation management of platypus populations is highly dependent on reliable demographic surveys of population sizes, particularly in small streams, where populations are low, and where connectivity with permanent drought refugia of larger streams may be vital for survival.

Using Capture-Mark-Recapture (CMR) surveys of a platypus population over 40 years (1973–2014), we modelled survival using Cormack–Jolly–Seber (CJS) framework, estimating age and sex-specific survival rates. We aimed to identify how survival patterns varied with demographics and river condition. We also identified key life history characteristics, including breeding and movement, allowing us to combine this information with survival estimates to produce a population dynamic model, quantifying the importance and uncertainties of key population vital rates, indicative of long term viability of platypus populations.

## Methods

### Study site

**Figure 1:**
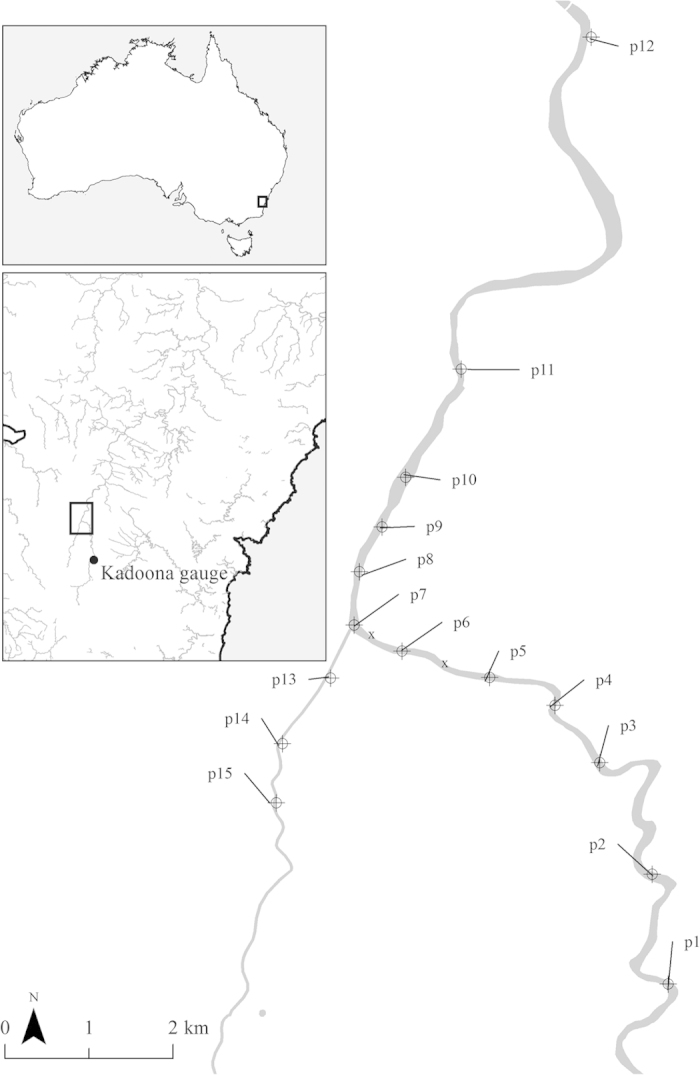
Location of the extant 15 pools (p) in the Shoalhaven River (1–12) and Jerrabattgulla Creek (13–15) (shaded) and two filled-in pools (x) in south-eastern Australia (insets), where platypus were captured and marked, 1973–2014. River flow was measured at the Kadoona flow gauge. Figure was generated using ArcGIS 10.3^95^.

We sampled the platypus population in 15 pools, separated by riffle areas, in 12.5 kilometres of the upper Shoalhaven River and 3.9 kilometres of the adjacent Jerrabattgulla Creek on the tablelands of southeastern Australia (Fig. 1). Some pools could not be sampled from the early 1990s because they in-filled with sand deposition (‘sand slugs’), no longer providing suitable foraging habitat and becoming unsuitable for our capture methods. Platypuses were captured using the unweighted mesh (“gill”) nets^30^ and individuals were initially (<1987) marked with stainless steel leg bands^30^ but later injected with Passive Integrated Transponder (PIT) tags^31^. In total, we deployed survey net for over 5,600 hours. Surveys were predominately carried out over the spring and summer months (December–March) to align with the breeding and juvenile emergence period (72% of net hours and 82% of capture records). December and March had the largest proportion of net hours (32% and 23%) and of captured platypuses (30% each).

Each individual trapped was sexed, aged and weighed. We determined sex from the presence (male) or absence (female) of calcaneal spurs^32^,^33^. Lactating female animals were identified by injecting 0.2 mL of synthetic oxytocin (2 IU, Syntocinon, Sandoz) intramuscularly and squeezing the mammary glands for milk^34^. We calculated the annual lactation likelihood of breeding female platypuses as the ratio of lactating and non-lactating females, captured during December-January, a period when females were most likely to lactate^22^. Absolute ages were determined primarily through recaptures and observation of respective loss or morphological change in the female vestigial spur sheath or male spur^32^,^33^. We had three immature categories: females with a spur sheath present in their first year (juveniles), juvenile males (age ≤1) and sub-adults males (age 1–2). All other individuals were categorised as adults. Females lost their spur sheaths between October and December in their first year, after emerging from nesting burrows, and were categorised subsequently as adults. Contrastingly, males developed full adult spurs by their third year of life. Subsequent recaptures permitted minimum ages to be assigned to individuals, not initially caught as juveniles, beginning with a minimum age at first capture of one year for adult females and two years for adult males.

We examined differences in weight and length between each of the sexes and age classes. Multiple recaptures of individual adult platypuses were only considered once by estimating the overall average. Recaptures of juvenile platypuses were also considered once, unless recaptures extended into adulthood where we considered an average for the juvenile stage and an average for the adult stage. We used a Bayesian linear model: y_i_ = βx_i _+_ _e_i_; where y was the response variable (weight or length), x the five age\sex classes, β a vector of the coefficient and, e the error term. Errors were assumed to be Gaussian, with mean zero and constant variance. We used semi-conjugate priors; a multivariate Gaussian prior for the slope coefficients and an inverse Gamma prior on the conditional error variance. A standard non-informative prior was used for the coefficient term (mean and precision equal to zero), as well as on the conditional error variance (c_0_\d_0_ equal to 0.001). The Markov Chain Monte Carlo (MCMC) algorithm (i.e, Gibbs sampling) was implemented to approximate the joint posterior distribution. To ensure convergence, 10,000 draws were taken after an initial 1,000 burn-in draws and a thinning interval of five. MCMC convergence was checked using a trace plot and a density plot. We performed the Bayesian linear model, using the MCMCpack package^35^, within the R statistical software^36^. We evaluated variation in weight of platypuses across the four decades: 1973–1983/1984–1993/1994–2003/2004–2014. For multiple recaptures of individual platypuses, we estimated their average weight per decade. We also evaluated seasonal changes in weight by estimating the average weight of adult female and males platypuses for each month. We considered recaptures of animals within the same month once by calculating the average weight.

**Figure 2:**
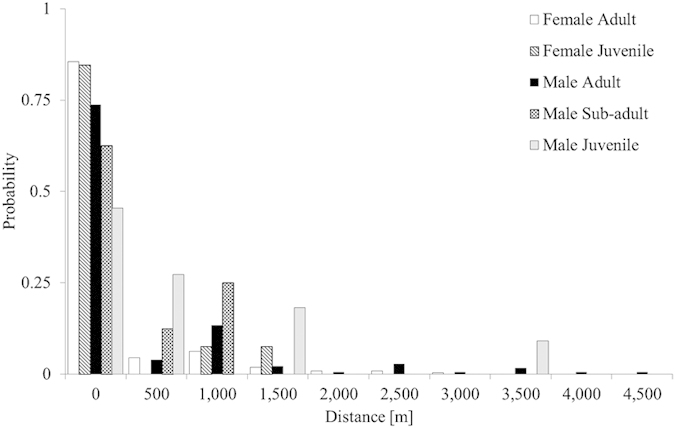
Distances moved by individual platypus, between the 15 surveyed pools (Fig. 1), in five age and sex classes: adult female (FA− white filled), juvenile female (FJ-diagonal lines), adult male (MA-black filled), sub-adult male (MSA− grey checker board), juvenile male (MJ− light shaded).

To evaluate decadal trends in the proportion of lactating females and proportion of female platypuses and the proportion of recaptures for each of the sex and age classes, we used a Bayesian test of proportion, using the BayesianFirstAid package^37^, within the R statistical software^36^. This estimated the relative frequency of lactating females or female platypuses (θ) for each of the four decades (n), (1973–1983/1984–1993/1994–2003/2004–2014) or the relative frequency of recaptures (θ) for the five classes (n), (juvenile and adult females; juvenile, sub-adult and adult males). Individual females were considered once per year and multiple times within each decade. The Bayesian model assumes: x ~ Binomial(θ, n) and θ ~ Beta(1, 1). We also examined whether total annual cumulative river flow volume was related to the ratio of lactating females, using a Bayesian Poisson regression model, according to earlier assumptions and processes. Flow volumes were measured at the Kadoona gauge on the Shoalhaven River (http://realtimedata.water.nsw.gov.au), about 20 km upstream (Figs 1 and 2, Appendix 1). We also estimated the likelihood of consecutive years of breeding by comparing the proportion of females lactating or not lactating in any two consecutive years.

We estimated movement rates of recaptured platypuses in the 15 pools separately for the two sexes and age classes. To evaluate the likelihood of differences among sex and age class in the proportion of residency vs. movement, we also used a Bayesian test of proportion with similar assumptions. We also examined differences in movement (distances travelled) between sex and age class using a Bayesian linear model following similar assumptions and processes as stated earlier.

### Determinants of survival and recaptures

We estimated survival and recapture probabilities, based on MARK^38^, using the RMark package^39^ in R^36^. Probabilities of apparent survival (Φ) and recapture (p) were derived using Cormack-Jolly-Seber modelling^38^,^40^. Apparent survival do not represent probabilities of true survival as mortality and emigration could not be distinguished without knowledge of individual movements^41^. Nonetheless, apparent survival is useful for comparing estimates between studies using mark-recapture monitoring techniques as well as to identify possible factors affecting animal survival. To estimate potential factors affecting survival, we also modelled relationships between survival estimates and sex, age class (0–1, 1–3, ≥3), weight, and the cumulative river flow (GL) over the previous 1, 6, 12, 24 months before capture date. We also examined the maximum total monthly flows between January and December and maximum total monthly flows between January and April, the period aligning with the platypus’ breeding and emergence of young. High stream flows can reduce the availability of macroinvertebrate prey species^24^, increase metabolic demand on foraging platypuses^42^ and drown dependent nestlings in burrows during the breeding season^22^,^24^.

To model relationships with recapture probabilities, we included cumulative flow over the month of sampling and sampling effort. Our use of unweighted mesh nets meant that during moderate to high flow conditions, nets could be lifted by the current, allowing platypuses to swim underneath, affecting recapture rates. We defined our survey effort as the total annual number of hours nets were in the water. Annual survey effort was quantified for each survey and pool combination, corresponding to the captured and marked platypuses. For model selection, we used an information-theoretic approach with Akaike’s Information Criterion, corrected for small sample size (AICc), to control for model parsimony, allowing statistical inferences^38^,^40^. We assumed surveyed platypus population was a single open population. We estimated annual apparent survival and recapture rates using a model averaging approach and considered all top ranking models with a cumulative weight of 99%. Parameter estimates from each model were weighted using the AICc score for that model.

### Population dynamics and viability

We modelled demographics, probability of extinction and growth rates of platypus populations, using an individual-based simulation of the VORTEX software, Version 9.15^43^,^44^. We parameterised our population models, using available life history data. Apparent survival estimates from live Capture-Mark-Recapture (CMR) can be negatively biased due to permanent emigration\dispersal of marked individuals from a study area^45^. We explored these effects on population viability by discounting dispersal rates, using our estimated residency rates, and deriving an adjusted measure of apparent survival for each sex and age class, more realistically representing true survival rates. This adjusted survival rate of age/sex class y (S_y_) was derived from apparent survival rate (Φ_y_) and residency rate (r_y_): 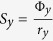.

We examined uncertainty of input variables in our population viability model, using sensitivity analysis^46^. This included incorporating full likelihood ranges of dispersal success for all dispersing individuals (0–100%) and annual mortality rates (0–100%) for the five sex and age class. To identify potential thresholds in the response of extinction probabilities, we used a flexible approach, relying on a generalized additive model (GAM). Here, the predictor depended linearly on unknown smooth functions of some of the covariates^47^: y_i_ = s(x_i_) + e_i_; where y was the log-transformed time to extinction; x was the predictor variable; s() was the smoothing function and; e was the error term. To avoid over fitting, the smoothing parameter was estimated using the generalized cross validation criterion, implemented using the mgcv package^48^,^49^, within the R environment^36^.

## Results

### Life history

Eight hundred and twelve unique platypuses were captured and marked (1973–2014); there were 810 recaptures of these, for a total of 1,622 captures. These individuals included 348 adult females, 157 juvenile females, 165 adult males, 41 sub-adult males, and 100 juvenile males. One female and one male were respectively recaptured over 21 and 8 years. Annual frequencies of lactating females during peak breeding season (December–January) ranged between zero and 15 animals (average 4.83 ± 3.50sd), converting to an average probability of 0.39_ _±_ _0.15sd of platypus females in the population lactating in a breeding season. The probability that a given female would be lactating each year was *P* = 0.37 ± 0.44sd (ratio of lactating and not lactating in any given year when captured during December–January), an index of breeding rates. The maximum age of a lactating female was 21 years, although this was only an observed limit. There was no decadal trend in the proportion of lactating females (*P* = 0.40[95% Credible Interval: 0.28–0.52] (1973–1983), 0.42 [0.33–0.52] (1984–1993), 0.38 [0.27–0.49] (1994–2003), 0.31 [0.19–0.43] (2004–2014)). There were also no relationships between probability of lactation and total annual flow volumes in the current (β = 0.09 ± 0.36se) or preceding year (β = 0.24 ± 0.36se). Over the end of the breeding season (February–March), lactating platypuses had slightly lower average weight (852.8 ± 30.2sd) compared to that of non-lactating platypuses (877.3 ± 87.1sd). Of all platypuses tested for lactating in any two consecutive years, (n = 127), 13% lactated in both years, 20% lactated in only the first, 19% in only the second, and 47% did not lactated in either.

There was a significantly skewed sex ratio in favour of females over males, consistent over the four decades: *P* = 0.60[95%CI: 0.55–0.65], 0.65[0.60–0.71], 0.64[0.60–0.71], 0.70[0.62–0.77]. Although there was no obvious trend in ratio of juveniles to adults over three decades (*P* = 0.29[95%CI: 0.25–0.34], 0.28[0.23–0.33], 0.35[0.28–0.43], 0.19[0.13–0.25]), the ratio in the last decade (2004–2014) was significantly lower compared to the previous three decades (Δ*P* = *−*0.1[−0.18–−0.02], −0.09[−0.17–0.003], −0.16[−0.26–−0.07]). Proportion of captured juvenile females increased over the spring and summer months, peaking in March (*P* = 0.22) and April (*P* = 0.24). High proportion of juvenile female platypuses were also recorded in September (*P* = 0.16) and October (*P* = 0.13). Juvenile males had high proportion between February and April (*P* = 0.16, 0.12, 0.13, respectively) but also peaked in September (*P* = 0.10). Adult females (mean 860.0 g) were significantly heavier than juvenile females (658.4 g). Similarly, adult males (1375.6 g) were significantly heavier than sub-adults (1189.8 g) and juvenile males (825.8 g), and all female classes (Table 1). There were similar significant differences in length, among the five sex\age classes (Table 1). Seasonal variation in weight was observed in both female and male platypus. Adult female platypuses were observed to maintain similar and high weight during the mid-summer to early autumn (December to April) with an average weight of 873.1 g ± 4.5sd. Weight of adult female platypuses steadily decreased to a minimum average between July and September with an average weight of 756.6 g ± 22.2sd. Similarly, adult male platypuses were the heaviest between November and March, with an average weight of 1400.2 g ± 30.4sd. Generally, adult male platypuses decreased in weight, reaching a minimum between August and October with an average weight of 1246.9 g ± 42.1sd.

### Mark-recapture and survival

The number of times individual platypuses were captured (i.e., capture rate), over 40 years, ranged between 1 and 21 (average 1.56[95%CI: 1.47–1.64]). Adult and juvenile female capture rates were higher (1.77[1.64–1.90] and 1.56[1.37–1.75], respectively), compared to adult and juvenile male capture rates (1.42[1.23–1.60] and 1.18[1.00–1.39], respectively). Most platypuses were only captured once with juvenile platypuses recaptured significantly less frequently than adults (recapture female adult: *P* = 0.40[95%CI: 0.35–0.45], female juvenile: 0.22[0.16–0.28], male adult: 0.29[0.22–0.36], male sub-adult: 0.35[0.22–0.50], and male juvenile: 0.06[0.02–0.11]). These represented significant differences in recapture rates between adult and juvenile females (Δ*P* = 0.18[0.01–0.26]), adult females and adult males (Δ*P* = 0.11[0.03–0.20]), and between juvenile males and all other groups, including juvenile females (Δ*P* = 0.16[0.08–0.24]).

Apparent survival varied with sex, age class and weight (top models with 99% of the overall weight, Table 2). Over the 40-year survey period, there were strong sex and age differences in apparent survival estimates, with significantly higher survival of adult females (Φ = 0.76 ± 0.05sd), compared to adult males (Φ = 0.57 ± 0.06sd), (Table 3, Appendix 2). Concurrently, apparent survival of juvenile females (Φ = 0.27 ± 0.04sd) was significantly higher than for juvenile males (Φ = 0.13 ± 0.02sd) and similar to sub-adult males (Φ = 0.38 ± 0.05sd), (Table 3, Appendix 2). For adult females (max 1,150 g, Table 1), weight was continuously positively related to apparent survival estimates, peaking at Φ = 0.88 (Appendix 3). For adult males (max 2,000 g, Table 1), there was a humped-shaped relationship between weight and apparent survival estimates, peaking around 1,300 g (Φ = 0.57), (Appendix 3).

In addition, flows were significantly related to survival estimates, with strongest support for cumulative flows in the previous six months, followed by cumulative flow in one month (ΔAICc = 1.49), and previous 12 months (ΔAICc = 6.34, Table 2). Cumulative flows were negatively related to survival of platypuses but predominantly at extreme high flows (Table 3, Appendix 2). For example, apparent estimated survival of adult females (average weight) was reasonably stable (Φ = 0.78–0.72), up to total flows of 60 GL over the previous six months but, significantly declined by about 27% to Φ = 0.57 when total flows were 120 GL (Table 3, Appendix 2). All models included sampling effort and cumulative flows in the month during capturing as determinants of capture probability (Table 2). Under long-term average cumulative flow conditions during trapping (3.8 GL), probability of detection of adult platypuses was predicted to increase from *p* = 0.3 to *p* = 0.8 as total net trapping hours increased from 10 to 180 (Table 2, Appendix 4). Age was also a factor influencing detection probabilities with some support that sex also affected detection (ΔAICc = 1.95, Table 2). Juvenile platypuses were detected (i.e., trapped) more easily, with detection probabilities increasing from *p* = 0.47 to *p* = 0.89, as net hours increased (Table 2, Appendix 4).

### Movement

Based on recapture records in the 15 pools, we estimated distances moved for the two sexes and different age classes, over 40 years (Fig. 2). Most adult and juvenile females were likely to remain in the same pool (*P* = 0.85[95%CI: 0.82–0.88] and 0.93[0.83–0.99], respectively). Residency rates were significantly higher for adult male platypuses (*P* = 0.74[0.67–0.80]), than other male classes: decreasing to *P* = 0.66[0.47–0.83] in sub-adults and as low as *P* = 0.43[0.21–0.67] for juvenile males (Fig. 2). There were significant differences in distances moved between adult males and adult females (Δ*P* = 0.11[0.04–0.19]), and between juvenile females and sub-adult males (Δ*P* = 0.26[0.07–0.47]) and juvenile males (Δ*P* = 0.49[0.23–0.72]). There were also significant differences in the distances between recaptures of males and females. Mean distances travelled by adult females were significantly shorter (mean: 128.5 m [88.8–167.7]) than those travelled by adult males (310.1 m [234.6–386.5]), (Fig. 2). Juvenile males (493.8 m [245.0–748.1]) also moved considerably more than adult males, but not sub-adults (421.4 m [231.7–617.6]). The maximum distance between consecutive recaptures in females was 2.8 km. For males, this maximum distance was 4.2 km. Further, 2% of females and 7% of males had consecutive recaptures greater than 2 km apart (Fig. 2).

### Population dynamics and viability

Across all stream reaches of the study area (16.4 km), the total annual number of platypuses captured varied considerably over the 40 year period, ranging between 2 and 99 (average 30.5 ± 20.3sd). Based on derived detection probabilities using Mark-recapture models, and incorporating sampling effort and environmental conditions, annual platypus numbers ranged between 5 and 107 (average 46.7 ± 21.5sd), representing an annual trend of −0.53. In the study reach more consistently sampled (~2 km; pools p8, p9 and p10; Fig. 1) the number of platypuses ranged from 2 to 42 (average 18.2 ± 9.4sd), with adjusted numbers varying between 5 and 63 (average 28.9 ± 12.9sd), representing an annual trend of −0.17.

**Figure 3:**
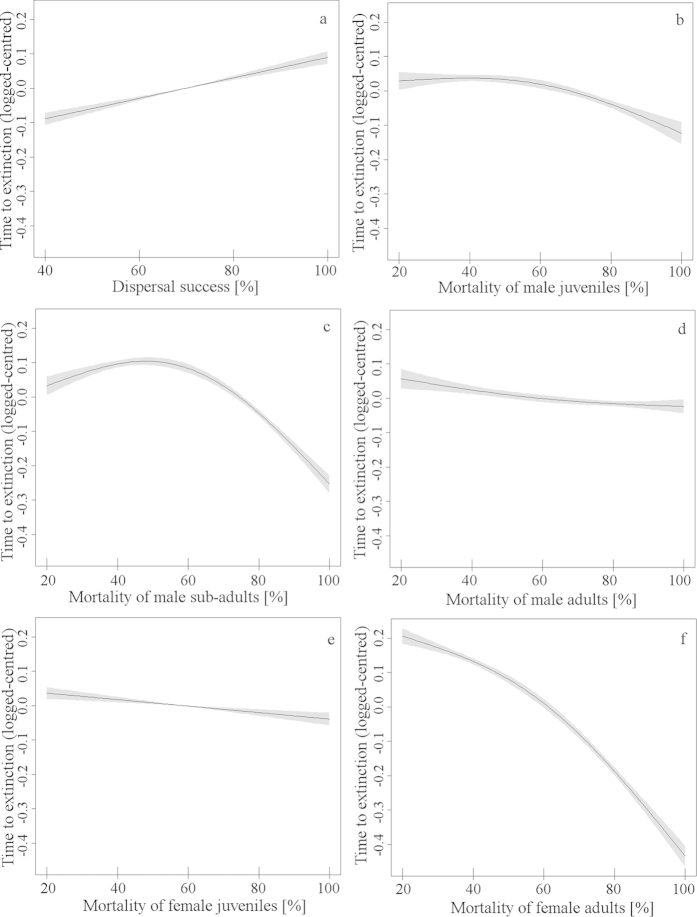
(**a**–**f**) Mean (±2SE) time to extinction (centred and log-transformed) relative to key population parameters: (**a**) dispersal success rate [%] and annual mortality rates [%] of (**b**) juvenile male, (**c**) sub-adult male, (**d**) adult male, (**e**) juvenile female and, (**f**) adult female, (see Table 4). Relationships were based on sensitivity analyses from Vortex population viability analyses and modelled, using Generalized Additive Models.

Using estimates of apparent survival, population viability was extremely low, with a predicted negative mean population growth rate (r = −0.308 ± 0.130sd), estimating extinction within 20 years. Using adjusted survival rates, based on estimated residency rates (i.e., dispersal, Table 4), there was evidence that the population was stable, with a mean population growth rate of r = 0.01 ± 0.07sd, and the likelihood of extinction in 100 years only 4%. We explored uncertainty of our survival estimates and dispersal success with sensitivity analyses, comparing extinction probabilities across a range of survival and dispersal rates (Fig. 3 and Table 5). This highlighted key population parameters, critical for long term viability. Survival of adult females had the greatest impact on maintaining population viability, achievable only when their survival rates were above 0.55 (Fig. 3 and Table 5). Contrastingly, viable populations were achieved when survival rates of males (juvenile, sub-adult, and adult) and juvenile females were as low as 0.1. However, these were obtained only when survival rates of dispersing animals were above 0.35, a key requirement to maintaining viable populations, after survival of adult females (Fig. 3 and Table 5).

## Discussion

This 40-year study of over 812 platypuses is the longest continuous study on the life history and population dynamics of this iconic and notoriously difficult to investigate species. Through our Capture-Mark-Recapture modelling, we identified strong sex bias in occurrence and life history, including morphology (weight and length), movement and longevity. The population was dominated by females (*P* = 0.60–0.70), with no significant trend in number of females over 40 years. There were similar sex ratios for adult females in a southern Victorian creek^24^, but juvenile and adult sex ratios vary elsewhere, including no significant difference from parity to significant male bias^1^. Yet, variable capture probabilities may act as a possible confounder of sex and age ratios in surveyed platypus populations, particularly with noted lower capture probabilities of juvenile platypuses (Table 3). Lower capture probabilities of adult males and juvenile females are known to occur^22^,^50^. Whether sex ratios represent adaptive life history strategies in mammals, indicative of resource availability, remains unclear^51^.

Morphological dimorphism, with considerably larger males (around 12–16% longer and 35–40% heavier; Table 1), is well recognised in platypuses^1^,^52^. Many mammal species are similarly dimorphic where males may need to compete for females^53^. Typical sexual dimorphism occurred in our study population, with no long-term variation over the four decades. A clinal variation of weights and lengths in the platypuses, increasing from low to higher latitudes, is well documented^16^,^23^,^52^,^54^. Platypuses from north Queensland are the smallest (mean lengths and weights for adult females and males): 37.7 cm  ± 3.1sd/737 g ± 86sd and 43.6 cm ± 3.2sd/1118 g ± 197sd, respectively^16^, while those in Tasmania are the largest: 44.9 cm ± 4.0sd/1232 g ± 23sd and 54.8 cm ± 4.0sd/2154 g ± 33sd^23^. Sizes of platypuses in our upper Shoalhaven River population, in the lower third of its north-south distribution (35.5°S), fitted the cline of weight and length measures (Table 1).

Movement behaviour also varied considerably between sex and age classes, likely reflective of the mating system of the platypus, although considerable knowledge gaps still remain. Males, predominately juvenile males, were more likely to be captured in more distant surveyed pools (Fig. 2). Our study and that of Furlan *et al*.^14^ also indicated considerable philopatry in adult females. Platypus populations appear to be composed of resident and transient individuals^24^,^55^,^56^ with males occupying larger home ranges than females^12^,^25^. During the breeding season (late winter to spring), the male’s venom glands, connected by ducts to the spurs, increase in size and output which coincides with elevated male aggression^1^,^33^. During this period, spatial and/or temporal separation forms in males^57^,^58^,^59^ which may compete for access to females, potentially exhibiting a polygynous mating system^1^,^32^,^33^.

Similarly, there was a strong bias in apparent survival, varying between sex and age classes. Adult females had a higher (33%) annual apparent survival estimate, compared to those of adult males (Table 4, represented as mortality rates: 100%-survival). Annual survival of juvenile males was half of that estimated for juvenile females, on the basis of apparent survival (Table 4). Apparent survival estimates also included permanent emigration^60^,^61^,^62^, not precluded from using Capture-Mark-Recapture surveys and the Cormack-Jolly-Seber (CJS) modelling^63^,^64^,^65^. Thus, some of the low survival estimates for juvenile platypus males were probably because most juvenile male platypuses dispersed quickly from the population^66^. Adjusting apparent survival estimates, using residency estimates, increased annual survival rates, particularly for juvenile males (Table 4) and suggested more stable populations. This dispersal, although incurring increased mortality, connects populations in rivers and likely reduces kin competition and inbreeding, as in most mammals^67^,^68^. Dispersal of juveniles can also be influenced by environmental conditions and resource availability^69^,^70^ as well as population densities^71^. Although survival estimates of dispersing platypuses are presently unknown, sensitivity analysis indicated this to be a significant component of population viability.

The only other survival estimates for different demographic classes of platypuses come from streams in Melbourne, Victoria^24^. Our survival estimates for juveniles (<2 years), adjusted for dispersal, were considerably lower (−60% females, −76% males) than those of platypuses in Melbourne streams but were higher for adults (+40% 3–5 years, +83% 6–8 years). A possible reasons for these differences may be attributed to trapping methods; here unweighted mesh (“gill”) compared to weighted fyke nets used by Serena *et al*.^24^, resulting in different trapping efficacy, possibly varying with age^50^. Explicitly, capture efficacy of juvenile platypuses using fyke nets may be considerably higher compared to that of unweighted mesh. While platypus populations in Melbourne were estimated to be approximately 1 platypus per km of stream, we had 2.8 platypuses\km, across the entire study area and 19.3 platypuses\km in the more consistently surveyed pools (p8–p10, Fig. 1). On Kangaroo Island, there is considerable variability in platypus estimates, ranging between 1.3 and 3.6 platypuses\km^72^ and 4–12 platypuses\km^73^. However, robust estimates of population size remain elusive^21^. Variation in population estimates could be due to choice of survey technique, sampling season^1^,^23^, habitat availability^74^,^75^ or unexplained variability in capture rates^76^,^77^,^78^.

Inevitably, robust estimates of survival and viability depend on obtaining more accurate estimates of movements and dispersal^45^,^79^, particularly for the platypus given their amphibious, cryptic, and mainly nocturnal behaviour^1^. Genetic analyses have identified gene flow between populations, particularly between adjacent river systems^6^,^7^,^14^,^80^, inferring historical movement. However, there are few data available indicating the nature of current movement within and between rivers and river systems. Radio-tracking and tagging is constrained by battery size and life, difficulties in long-term attachment of devices and recapture for retrieval^81^. In-stream readers/recorders for Passive Integrated Transponder tags (microchips) have short detection range (<1 m)^82^ and, with acoustic tags, cannot detect animals out of water^81^. Global Positioning System tags may provide some opportunity to improve estimation of survival and population dynamics.

We also tested the effects of flow magnitude and survey effort on capture success, affecting survival estimates. There was an inverse relationship between high flow volume and capture success, probably partly because platypuses can swim under unweighted mesh nets, lifted off the bottom by these flows. This did not explain how this relationship was also present when considering cumulative flow in the previous six months, rather than immediately post flood (1 month), (Appendix 4). Several potential factors could have contributed. In December-January of 1991/1992, there were several short high flood peaks when about half the female platypuses would have been feeding dependent offspring in nesting burrows (proportion of lactating females, *P* = 0.57, cf average *P* = 0.40 ± 0.17) but no newly-emerged juveniles were subsequently captured^76^ (Appendix 1). A similar event resulted in poor recruitment in a Victorian population^24^. Such flood levels can drown nestlings in burrows and inflict metabolic stress on foraging platypuses^42^. Also, distribution and numbers of platypuses in peri-urban streams around Melbourne were affected by the area of non-absorbent surfaces in their catchments and subsequent high run-off during rain events^83^. Floods can also reduce short term availability of macroinvertebrate prey although medium to high flow increase this productivity^75^.

The strong dispersal signal for males, particularly juvenile males, has significant implications for the potential impacts of river regulation on platypus populations. Low residency estimates of juvenile male platypuses, along with dispersal success, probably reflect the importance of movements and connectivity in maintaining population viability^84^. Rivers all have a unique dendritic spatial structure, posing considerable constraints on the population dynamics of aquatic obligate vertebrates^85^, affecting their abundance, distribution and metapopulation structure^85^,^86^,^87^. Habitat connectivity defines the spatiotemporal stochasticity of local populations, dampening declines and extinctions, and ultimately determining long-term persistence^88^. Maintenance of within-stream and overland dispersal of riverine vertebrates, especially juveniles, can significantly improve population persistence^84^. As there may only be a single colonisation path within and between rivers, fragmentation of rivers by dams and other structures may significantly reduce persistence^89^. The overland distance between tributaries and river systems and the range of in-stream structures, including large dams, represent significant barriers for platypus movements^16^. These probably contributed to distinctive genetic differences between platypus populations separated by barriers to movement^14^,^16^,^80^. Even water extraction may fragment rivers, affecting connectivity during dry periods, when large permanent refuge pools are essential for local population survival and breeding by providing the source recolonization. Projected increasing climate change will further challenge persistence of platypus populations^90^. Land use may similarly affect such refugia through erosion of banks and deposition of sediment (‘sand slugs’) which remove pools^15^, as occurred in our study. The building of new dams or increase in diversions for irrigation and other water uses will continue to fragment platypus populations, increasing short-term extinction risks for isolated populations, and threatening the long-term viability of the species.

### Implications

The platypus is a notoriously difficult species to investigate, particularly in the wild, but understanding of it ecology is increasing, particularly with data from long-term studies, such as ours. Our study also raises the question of applicability of population viability analyses across the range of the platypus. Declining local populations and the recently updated ‘near-threatened’ conservation status are fuelling demands for a national risk-assessment for the species^20^,^21^. Such an approach could be implemented across Australia, providing the first risk assessment, a national priority for the species, and also highlighting critical data required for management. Metapopulation analyses, incorporating threats, would help identify critical data required to test viability of different platypus populations as well as assessing risk to the species, even in the absence of a strong dataset across the range. Increased understanding of the confounding effects of dispersal on survival estimates is critical to adequately estimate population sizes and viability. Computational and analytical advancements now permit robust and large-scale metapopulation dynamic modelling^91^, built on the riverine networks^92^. Such analyses, across the range of a species, are able to adequately assess risks and mitigating actions needed by governments^29^. Use of information, even if imperfect, about conservation values, threats, costs and efficacy of conservation actions is critical^93^, providing necessary modelling evidence for decision-makers^94^. Progress in our understanding of population dynamics and critical mechanisms for persistence, including movement and dispersal behaviour, will be critical for understanding vulnerabilities of this iconic platypus.

## Additional Information

**How to cite this article**: Bino, G. *et al*. Life history and dynamics of a platypus (*Ornithorhynchus anatinus*) population: four decades of mark-recapture surveys. *Sci. Rep*. **5**, 16073; doi: 10.1038/srep16073 (2015).

## Supplementary Material

Supplementary Information

## Figures and Tables

**Table 1:** Mean (95% CIs), [sample sizes] of weight (g) and length (cm) of five age/sex classes (adult females (FA), juvenile females (FJ), adult males (MA), sub-adult males (MSA), juvenile males (MJ)), from captured platypuses overall and over four decades from the Shoalhaven population.

Sex\Age^a^	1973–1983	1984–1993	1994–2003	2004–2014	Overall
Weight (g)					
FA	858.6(838.1–879), [148]	869.9(848.6–891.3), [137]	862.5(833.2–892.3), [72]	865.4(836.9–894.4), [74]	864.1(851.8–876.3), [431]
FJ	655.7(621.9–690.2), [53]	656.4(622.6–691), [51]	620.3(575.7–664.5), [31]	646.1(594–698.8), [22]	647.4(627.2–667.5), [157]
MA	1425.7(1396–1455.6), [69]	1406.3(1375.2–1437.6), [62]	1432.2(1383.4–1481.6), [26]	1313.7(1267.7–1361.3), [28]	1403.4(1384.5–1422.4), [185]
MSA	1261.5(1206.7–1316.6), [20]	1134(1063.4–1205.1), [12]	1289.6(1196.5–1385.5), [7]	1086(1002.2–1169.5), [9]	1200.8(1164.8–1238), [48]
MJ	843.4(806.3–879.8), [44]	849.4(800.8–899.4), [26]	737.6(688.7–786.8), [26]	802.5(674.9–925.2), [4]	815.9(790.9–841.5), [100]
Length (cm)					
FA	41.8(41.5–42.2), [119]	43.4(43.1–43.8), [133]	42.8(42.3–43.3), [71]	42.8(42.3–43.3), [72]	42.7(42.5–42.9), [395]
FJ	38.5(38–39.1), [53]	39.8(39.3–40.4), [51]	38.9(38.2–39.6), [31]	39.1(38.3–39.9), [22]	39.1(38.8–39.4), [157]
MA	50.1(49.6–50.6), [59]	50.8(50.3–51.3), [58]	50.6(49.9–51.4), [26]	49.3(48.5–50), [26]	50.3(50–50.6), [169]
MSA	48.6(47.7–49.5), [19]	48(46.9–49.1), [12]	49.4(47.9–50.9), [7]	46.7(45.3–48.1), [8]	48.2(47.6–48.8), [46]
MJ	42.2(41.6–42.8), [43]	44.1(43.3–44.9), [26]	42.7(41.9–43.5), [25]	43.6(41.6–45.6), [4]	42.9(42.5–43.3), [98]

Multiple recaptures of individual adult platypuses were only considered once by estimating the overall average. Recaptures of juvenile platypuses were also considered once, unless recaptures extended into adulthood where we considered an average for the juvenile stage and an average for the adult stage.

^a^juveniles <1 years, sub-adult males 1–2 years, adult females >1, adult male >2 years.

**Table 2:** Top (99%) models from Cormack-Jolly-Seber modelling, using 868 marked-recaptured animals, 1973–2014, testing the effects of life history variables (sex, weight, age class) and cumulative flow (with increasing lags of one (1 m.Flow), six (6 m.Flow) and 12 months (12 m.Flow), ‘Kadoona gauge’, Fig. 1) before capture, on apparent survival (Φ) and detection probability (p).

Model^a^	#par	AICc	ΔAICc	weight	Deviance
Φ(6 m.flow + sex + weight + age)p(1 m.flow + effort + age)	13	2266.73	0.00	0.42	2240.44
Φ(1 m.flow + sex + weight + age)p(1 m.flow + effort + age)	13	2268.22	1.49	0.20	2241.92
Φ(6 m.flow + sex + weight + age)p(1 m.flow + effort + sex + age)	14	2268.69	1.95	0.16	2240.35
Φ(1 m.flow + sex + weight + age)p(1 m.flow + effort + sex + age)	14	2270.05	3.32	0.08	2241.71
Φ(6 m.flow + sex + weight + age)p(1 m.flow + effort)	11	2271.90	5.16	0.03	2249.68
Φ(6 m.flow + sex + weight + age)p(1 m.flow + effort + sex)	12	2272.98	6.24	0.02	2248.72
Φ(12 m.flow + sex + weight + age)p(1 m.flow + effort + age)	13	2273.07	6.34	0.02	2246.78
Φ(12 m.flow + sex + weight + age)p(1 m.flow + effort)	11	2273.36	6.62	0.02	2251.14
Φ(6 m.flow + sex + weight)p(1 m.flow + effort)	9	2273.88	7.15	0.01	2255.74
Φ(1 m.flow + sex + weight + age)p(1 m.flow + effort + sex)	12	2274.16	7.43	0.01	2249.91
Φ(6 m.flow + sex + weight)p(1 m.flow + effort + age)	11	2274.78	8.04	0.01	2252.56
Φ(12 m.flow + sex + weight + age)p(1 m.flow + effort + sex + age)	14	2275.00	8.27	0.01	2246.66
Φ(6 m.flow + sex + weight)p(1 m.flow + effort + sex)	10	2275.14	8.41	0.01	2254.97

Top models output included significant variables in the model; number of parameters (#par); the Akaike’s Information Criterion, corrected for small sample size (AICc); difference in AICc from top model (**Δ**AICc); the weight on the model based on AICc and; the deviance explained by the model.

^a^Weight and cumulative flows modelled as both single and quadratic components.

**Table 3:** Average estimated coefficients, standard errors and 95% credible interval from best models (ΔAICc ≤ 2, Table 2), relating apparent survival (Φ) and detection probability (p) to life history variables (age, weight, sex) and cumulative flow variables (cumulative flows in previous six months and one month), (see Appendices 2–4 for predictions).

Parameter	Covariate	Coefficient (β_i_) ± S.E.	95% C.I.
Apparent survival (Φ)	intercept^a^	−7.3804 ± 1.1808	−9.6947–−5.0661
	6 m.flow	0.0008 ± 0.0063	−0.0115–0.0131
	6 m.flow^2^	−0.0001 ± 0.0001	−0.0002–0
	male	−1.8673 ± 0.3059	−2.4668–−1.2678
	weight	0.0132 ± 0.0023	0.0086–0.0178
	weight^2^	0 ± 0	0–0
	sub-adult	0.3448 ± 0.4762	−0.5885–1.278
	adult	1.05 ± 0.3075	0.4472–1.6528
Detection probability (p)	intercept	−0.0417 ± 0.4132	−0.8516–0.7683
	1 m.flow	−0.0537 ± 0.0189	−0.0908–−0.0166
	effort^b^	0.0128 ± 0.0019	0.009–0.0165
	sub-adult	1.053 ± 1.0298	−0.9655–3.0714
	adult	−0.728 ± 0.4081	−1.528–0.072

^a^Intercept represents juvenile female platypuses.

^b^measured as total net hours.

**Table 4:** Model parameters and their sources, used for population viability analyses (Vortex software, Lacy 1993) of the platypus population on the upper Shoalhaven River.

Category	Parameter	Value (sensitivity analysis)	Source of life history information^a^
Dispersal settings	Min age at dispersal	1	Shoalhaven
	Max age at dispersal	15	Shoalhaven; Serena *et al*. 2013
	Sex biased dispersal	Both	Shoalhaven
	Dispersing adult males	26%	Shoalhaven
	Dispersing sub-adult males	38%	Shoalhaven
	Dispersing juvenile males	55%	Shoalhaven
	Dispersing adult females	14.50%	Shoalhaven
	Dispersing juvenile females	15.40%	Shoalhaven
	Survival of dispersers	100% (0–100%)	Unknown
Reproductive system	Type of mating system	Polygynous	Grant 2004
	Age of first offspring for females	2	Grant 2004
	Age of first offspring for males	2	Grant 2004
	Max age of reproduction	21	Shoalhaven
	Max (average) no. of progeny/year	2(1.5)	Grant 2004
	Males at birth	50%	Grant 2004
Reproductive rates	Females in the breeding pool	62%	Grant 2004; Serena *et al*. 2013
	Males in the breeding pool	38%	Grant 2004
Mortality rates	Female mortality ≤1yr (juvenile)	73%, (71%)^b^, (0–100%)	Shoalhaven
	Female mortality >1yr (adult)	24%, (11%)^b^, (0–100%)	Shoalhaven
	Male mortality ≤1yr (juvenile)	87%, (77%)^b^, (0–100%)	Shoalhaven
	Male mortality 1–2yr (sub-adult)	62%, (43%)^b^, (0–100%)	Shoalhaven
	Male mortality >2yr (adult)	43%, (23%)^b^, (0–100%)	Shoalhaven

^a^Shoalhaven refers to data collected during this long-term study.

^b^adjusted mortality estimates based on estimated residency rates.

**Table 5:** Coefficients of key life history parameters, relative to mean time to extinction, using Generalised Additive Models (all parameters were significant p < 0.001).

Variable	Demographic category	edf^a^	rdf^b^	F^c^
Dispersal	Likelihood of successful dispersal	1.446	1.693	44.27
Mortality	Female juvenile	1.588	1.83	11.41
	Mortality of female adult	1.916	1.993	133.64
	Mortality of male juvenile	1.78	1.951	15.04
	Mortality of male sub-adult	1.942	1.996	34.38
	Mortality of male adult	1.836	1.973	14.76

Mean time to extinction was estimated using population viability analyses (Vortex software, Lacy 1993). See Fig. 3a–f for predicted response curves.

^a^estimated degrees of freedom.

^b^reference degrees of freedom.

^c^F-statistic^49^.
