# Supplementary material for: Life history and dynamics of a platypus (Ornithorhynchus anatinus) population: four decades of mark-recapture surveys
Source: Sci Rep. 2015 Nov 5;5:16073. doi: 10.1038/srep16073 (PMC4633588; doi:10.1038/srep16073)

**Life history and dynamics of a platypus (*Ornithorhynchus anatinus*) population: four decades of mark-recapture surveys**

Gilad Bino^1,*^, Tom R. Grant^1^, and Richard T. Kingsford^1^

# Appendix 1

Cumulative flow (GL) estimates (1971-2014), measured at Kadoona Gauge on the Shoalhaven River (Fig. 1), incorporating different lags of 1 (dashed line), 6 (continuous line), 12 (dotted line), and 24 (continuous bold line) months, and sampling occasions (dots below x axis).


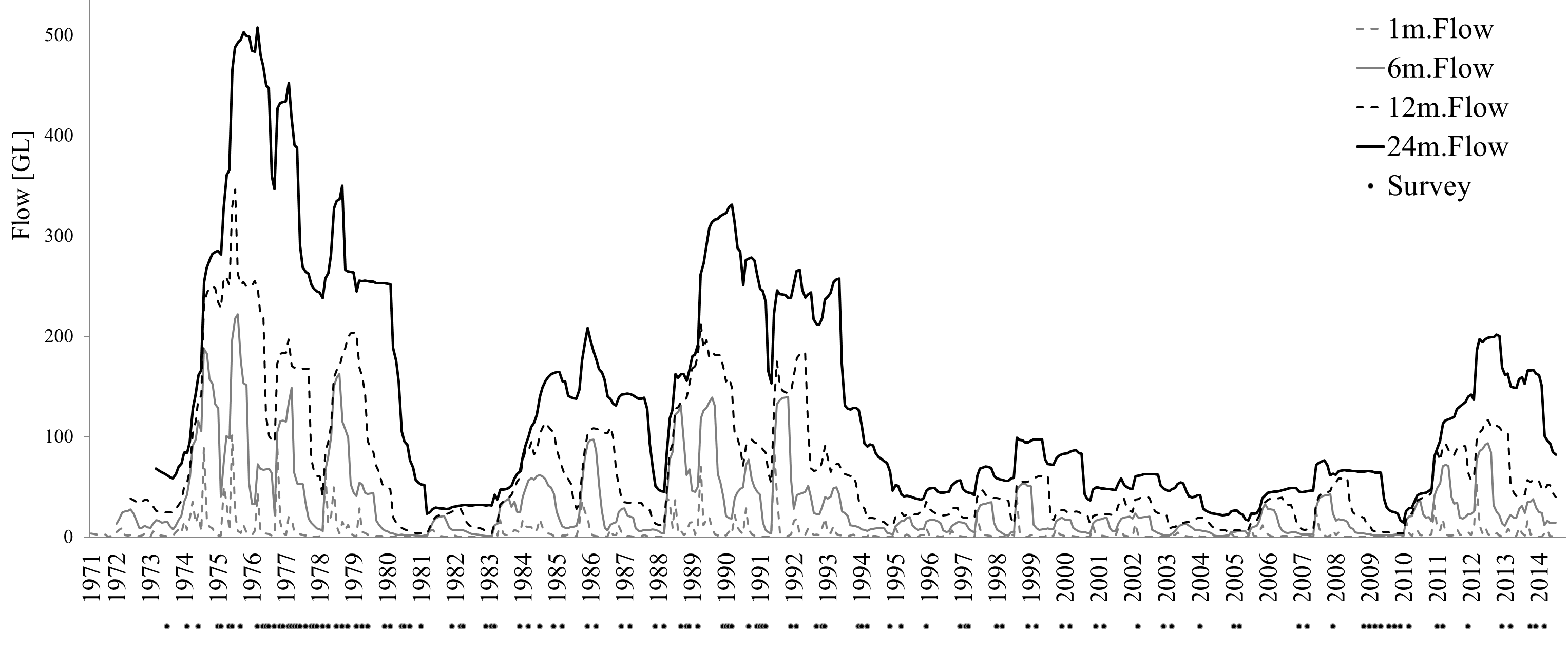


# Appendix 2

Predicted mean apparent survival rates of platypus for three life stages (adult, sub-adult, and juvenile), for males and females, relative to cumulative 6-month flow volume (GL), estimated using the Cormack-Jolly-Seber model from marked-recaptured animals (see Table 2). Unique average weight was used for the five categories.

Model:
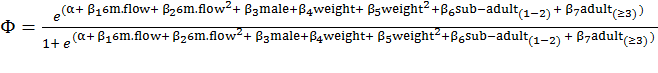


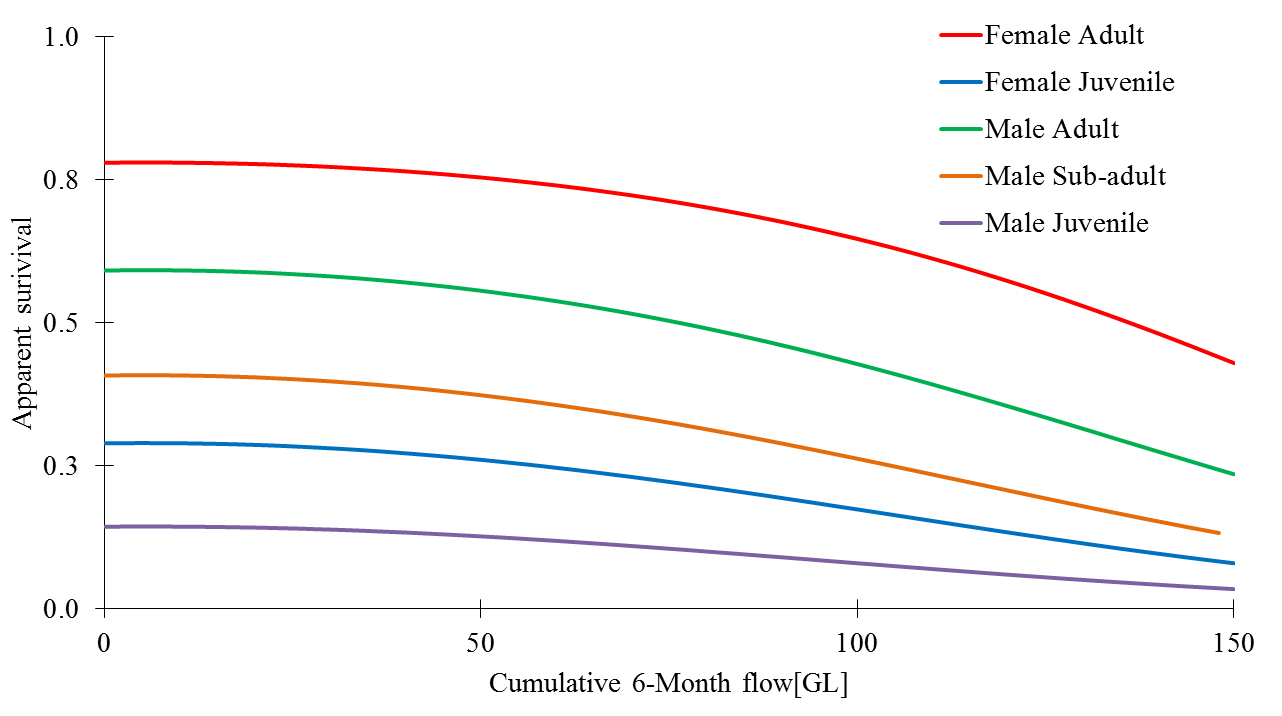


# Appendix 3

Predicted apparent survival rates of adult female (580 – 1,150g) and male (860-2,000g) platypus, relative to weight, estimated using the Cormack-Jolly-Seber model from marked-recaptured animals (see Table 2). Average cumulative flow in the previous 6 months was used (29.4GL).

Model:
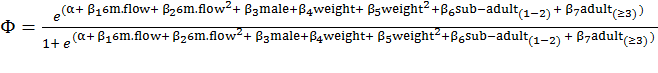


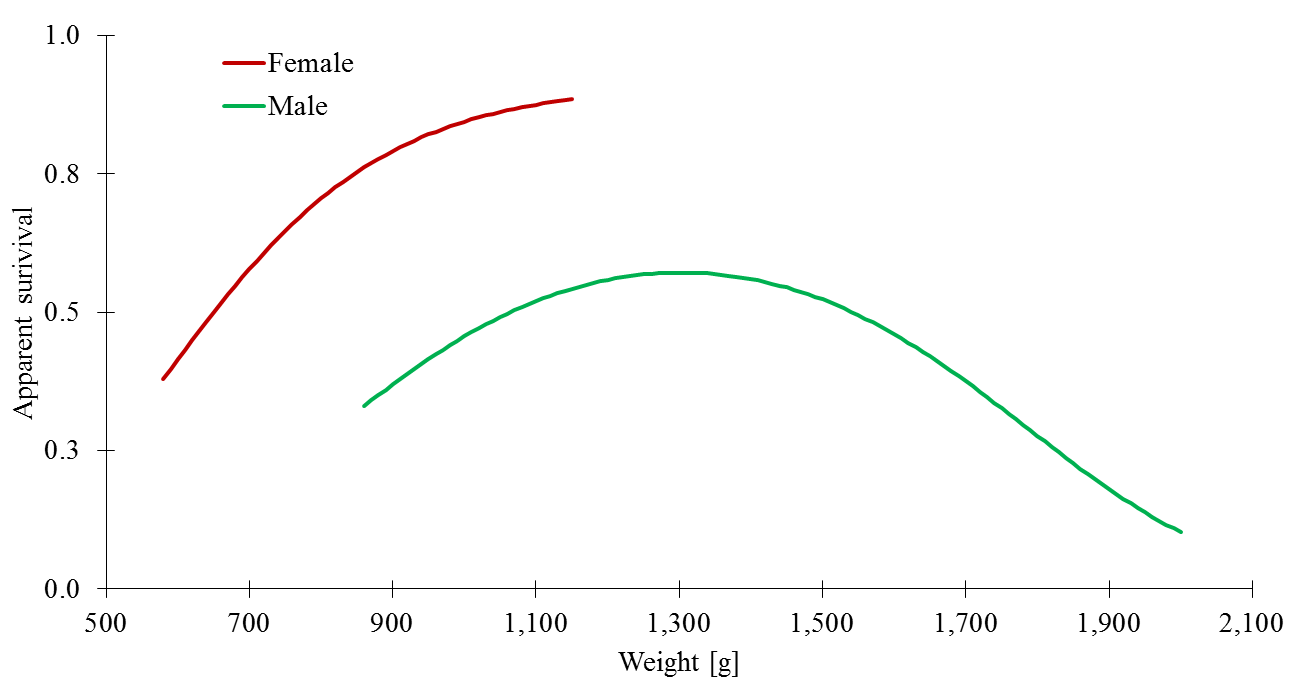


# Appendix 4

Predicted detection probabilities of adult and juvenile platypuses relative to annual sampling effort (net hour), estimated using the Cormack-Jolly-Seber model from marked-recaptured animals (see Table 2). Average cumulative flow in the previous 1 month was used (3.8GL).

Model:
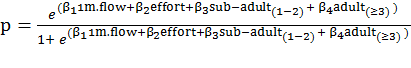


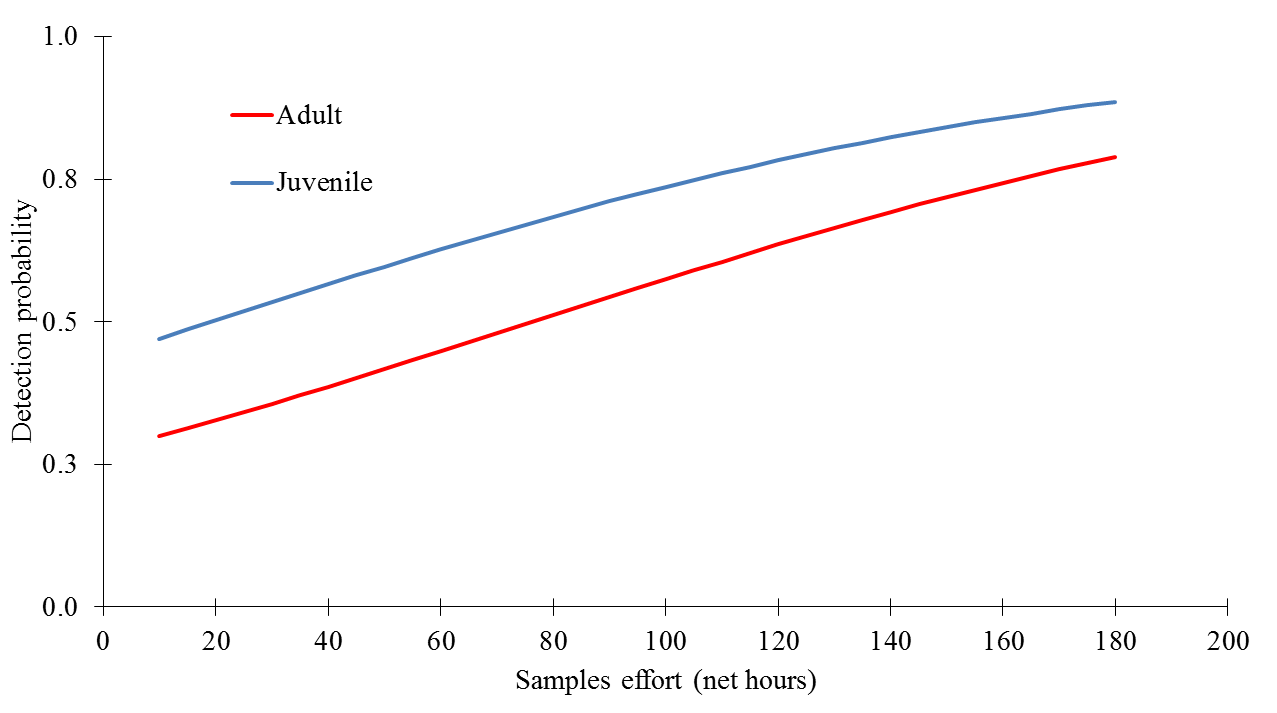


# Appendix 5

Predicted detection probabilities adult and juvenile platypuses relative to cumulative flows [GL] over the previous month, estimated using the Cormack-Jolly-Seber model from marked-recaptured animals (see Table 2). Average annual sampling effort (135 net hours) was used.

Model:
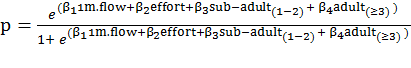


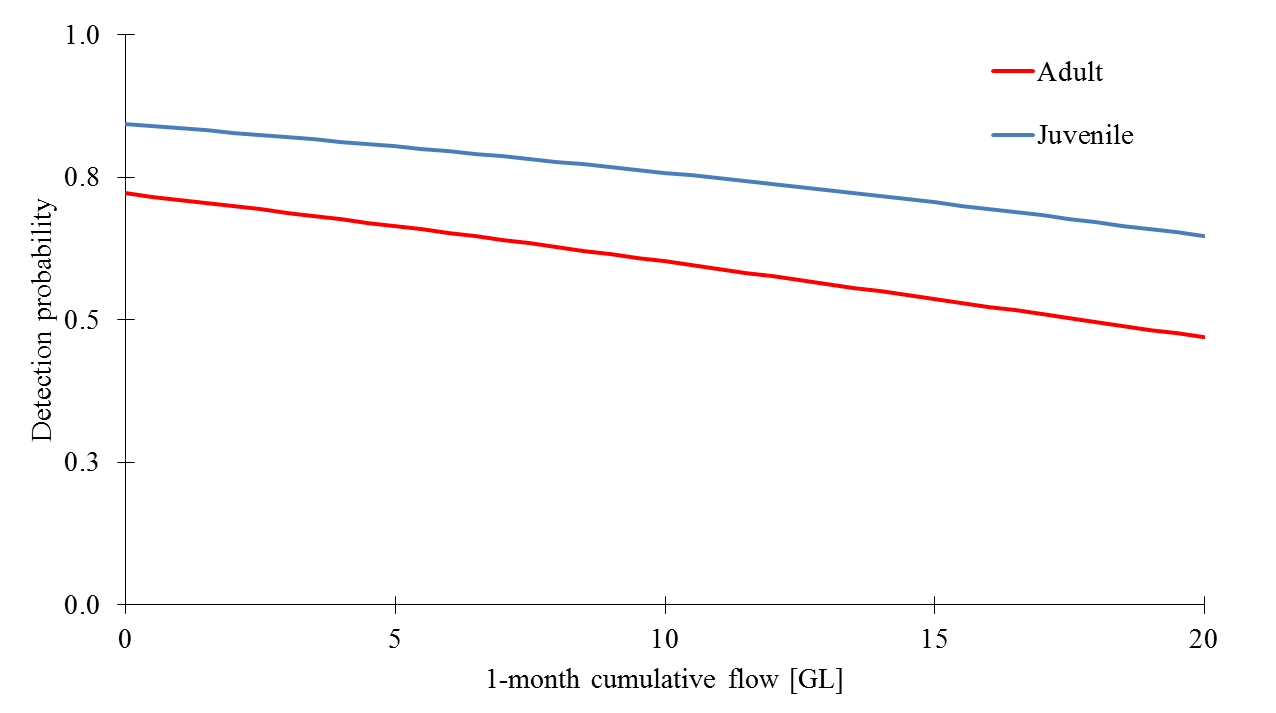

Supplement: Supplementary Information [file srep16073-s1.doc]
